# Analysis of Correlation Between White Matter Changes and Functional Responses in Post-stroke Depression

**DOI:** 10.3389/fnagi.2021.728622

**Published:** 2021-10-11

**Authors:** Xuefei Zhang, Yu Shi, Tao Fan, Kangling Wang, Hongrui Zhan, Wen Wu

**Affiliations:** ^1^Department of Rehabilitation, Zhujiang Hospital, Southern Medical University, Guangzhou, China; ^2^Department of Rehabilitation, The Fifth Affiliated Hospital of Sun Yat-sen University, Zhuhai, China; ^3^Rehabilitation Medical School, Southern Medical University, Guangzhou, China

**Keywords:** post-stroke depression, functional connectivity, structural connectivity, structural connectivity- functional connectivity coupling, resting-state fMRI, diffusion tensor imaging

## Abstract

**Objective**: Post-stroke depression (PSD) is one of the most common neuropsychiatric symptoms with high prevalence, however, the mechanism of the brain network in PSD and the relationship between the structural and functional network remain unclear. This research applies graph theory to structural networks and explores the relationship between structural and functional networks.

**Methods**: Forty-five patients with acute ischemic stroke were divided into the PSD group and post-stroke without depression (non-PSD) group respectively and underwent the magnetic resonance imaging scans. Network construction and Module analysis were used to explore the structural connectivity-functional connectivity (SC-FC) coupling of multi-scale brain networks in patients with PSD.

**Results**: Compared with non-PSD, the structural network in PSD was related to the reduction of clustering and the increase of path length, but the degree of modularity was lower.

**Conclusions**: The SC-FC coupling may serve as a biomarker for PSD. The similarity in SC and FC is associated with cognitive dysfunction, retardation, and desperation. Our findings highlighted the distinction in brain structural-functional networks in PSD.

**Clinical Trial Registration**: https://www.clinicaltrials.gov/ct2/show/NCT03256305, NCT03256305.

## Introduction

PSD is one of the most common mood disorders in stroke patients. A meta-analysis showed that the prevalence of major depression was about 18% (Meader et al., 2014). The main symptoms of PSD include negative emotions, cognitive deficits, and physical symptoms. Neuroimaging evidence from our studies and others has identified the structural and functional network disruption in PSD and other neurological diseases (He et al., 2009; Zhou et al., 2010; Zhou and Seeley, 2014). Recently, a graph-based approach was used in several studies to investigate the white matter connectivity between brain regions of PSD, which provided new insights into the SC and FC of these diseases (Yasuno et al., 2014; Chenfei et al., 2016). Compared with the healthy control group, increased path lengths and decreased local and global efficiency were observed in Alzheimer’s disease (Lo et al., 2010; Bai et al., 2012; Shao et al., 2012). Similarly, our previous studies found FC interruptions in PSD (Shi et al., 2017; Zhang et al., 2019). However, the topological attributes of the whole white matter structure and the relation between the structural and the functional brain networks in patients with PSD are still largely unknown.

Recent studies showed that SC-FC coupling values can quantify the consistency between SC and FC in combination with resting-state functional magnetic resonance imaging (rs-fMRI) and diffusion tensor imaging (DTI) data (Skudlarski et al., 2008). Moreover, SC-FC coupling has been applied for many other neurological diseases including idiopathic generalized epilepsy (Zhang et al., 2011) and schizophrenia (Van Den Heuvel et al., 2013).

The structural-functional relationship of large-scale brain networks may be disrupted in diseases (Hagmann et al., 2010; Wang et al., 2015). Both human and animal studies showed that the FC in health was similar to SC (Barttfeld et al., 2015). Compared with healthy people, the SC-FC coupling values were decreased in depressed patients, and the ability to process negative emotion might be more directly related to the SC abnormality (Liu et al., 2021).

Based on the fact that the brain can be isolated and integrated at the same time, it may not be enough to only study large-scale (whole-brain) SC-FC coupling. Therefore, recent studies also reported that the regional SC-FC coupling was reduced but there was no difference in SC-FC coupling at the whole brain level (Cocchi et al., 2014; Kim et al., 2019). These studies contributed to discovering the local characteristics of the brain network.

Modular structure helps to balance functional separation and integration, save path length (Sporns et al., 2000, 2007), process and exchange information efficiently (Latora and Marchiori, 2001; Kötter and Stephan, 2003; Sporns et al., 2007), and restore the disorders of network nodes or edge (Han et al., 2004; Sporns et al., 2007). Yi et al. (2015) found that increased inter-Module connections were related to cognitive score, so changes in functional Modules may enhance our understanding of PSD.

Here, we aimed to investigate: (i) whether the SC-FC coupling in PSD is different compared to non-PSD; (ii) whether SC-FC coupling is correlated to the course of PSD; and (iii) how the topological organization of the brain networks changes at different levels (including connectional, global and nodal properties).

## Materials and Methods

### Participants

**Figure 1:**
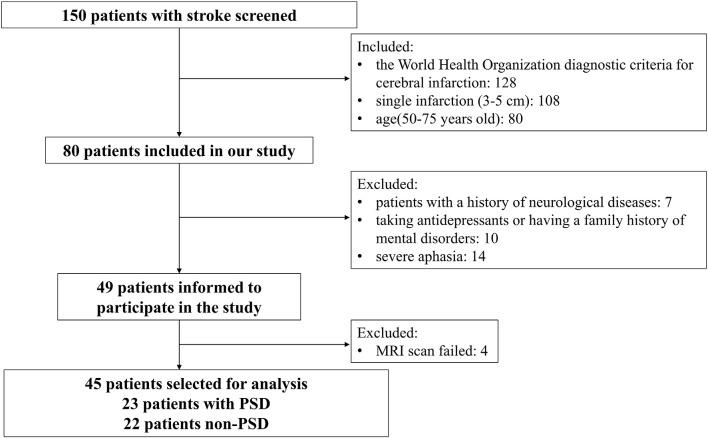
Flowchart of patient selection.

Twenty-three patients with PSD and 22 patients with non-PSD were recruited in this study (Figure 1). The inclusion criteria were as follows: (1) all subjects met the World Health Organization diagnostic criteria for cerebral infarction; (2) single infarction (3–5 cm); and (3) age: 50–75 years old. The exclusion criteria were as follows: (1) patients with a history of neurological diseases (such as epilepsy); and (2) taking antidepressants or having a family history of mental disorders. We collected the following information from each subject: demographics and the severity of stroke as measured by National Institutes of Health Stroke Score (NIHSS), Montreal Cognitive Assessment (MoCA), Mini-Mental State Examination (MMSE), and Barthel Index (BI). In the PSD group, participants met the Diagnostic and Statistical Manual of Mental Disorders, Fifth Edition (DSM-V) criteria for depression, with a Hamilton Depression Scale (HAMD) score of 17 or higher. Other participants were assigned to the non-PSD group. HAMD-24 was to assess the severity of depression. This study was approved by the Ethics Committee of the Zhujiang Hospital of Southern Medical University, China. All subjects signed informed consent.

### Brain Imaging

Experimental data were obtained with a Philips 3.0 Tesla magnetic resonance scanner (Royal Philips Electronics, Eindhoven, the Netherlands) equipped with a 64-channel head coil. Foam, helmets, and earplugs were used to reduce head motion and scanner noise. During the scan, all participants were asked to close their eyes and not to think. Sagittal high-resolution three-dimensional T1-weighted images were acquired using a magnetization-prepared rapid gradient-echo sequence: TR/TE = 2,000/2.26 ms; flip angle = 90; field of view = 256 × 256 mm^2^; in-plane matrix = 256 × 256; voxel size =1 × 1 × 1 mm^3^, no slice gap, 176 sagittal slices) for each subject. The structural sequence took 6 min. The DTI scan used single-shot echo-planar imaging (ss-EPI) (TR/TE = 12,500/112 ms; flip angle = 90°; field of view =230 × 230 mm^2^; in-plane matrix = 128 × 128; voxel size = 1.88 × 1.88 × 2 mm^3^, slice thickness 3.0 mm). Blood oxygenation level-dependent functional imaging was acquired using a gradient echo-planar imaging sequence as follows: (TR/TE = 2,000/35 ms; flip angle = 90; field of view = 230 × 230 mm^2^; in-plane matrix = 64 × 64; no slice gap; 33 axial slices, slice thickness = 2 mm). The fMRI sequence took 8 min.

### Data Analysis

We analyzed the network topology changes and used probabilistic fiber tracking based on 90 regions of interest. Based on the paired Pearson correlation between 90 regions of interest (Tzourio-Mazoyer et al., 2002), the graph theory global and nodal indicators are calculated for the two connected groups. Using the canonical 7-network parcellation given by Yeo et al. (2011) to define functional Modules: the visual, somatomotor, dorsal attention, ventral attention, limbic, and frontoparietal control Modules, and the default mode network.

### Construction of the Structural Network

According to the Anatomical Automatic Labeling (AAL) anatomical template, the brain was divided into 90 brain regions, and each brain region represented a node (Tzourio-Mazoyer et al., 2002). First, we preprocessed the DTI image based on previous research (Basser et al., 1994). Next, deterministic fiber tracking of the whole brain white matter in the individual space was performed based on the previous research (Yeh et al., 2013): the anisotropy threshold is 0.15, the angle threshold is 45°, all DTI preprocessing and fiber tracking are carried out in PANDA^1^. The edge of the structural network was defined as the number of fibers passing through the two nodes/the average number of voxels between the two nodes. We used SC to reflect the strength of the WM connection.

### Construction of the Functional Network

The resting state data was preprocessed by the Data Processing Assistant for Resting-State fMRI (DPARSF) toolbox (Chao-Gan and Yu-Feng, 2010). Preprocessing included head movement correction, registration to 3D T1WI, removal of covariates (head movement, WM signal, and cerebrospinal fluid signal), nonlinear spatial normalization using T1WI and band-pass filtering (0.01–0.1 Hz). Images with head movement exceeding half the voxel width (1.5 mm) were excluded from the study. After preprocessing, the average time series of each AAL area was obtained by averaging the voxel BOLD signals in the selected brain area (Tzourio-Mazoyer et al., 2002). The function network was defined as the Pearson correlation coefficient between the average time series of different nodes, and the function matrix was obtained.

### The Small-World (SW) Attribute

The SW attributes were mainly defined by the ratio of the clustering coefficient Cp to the path length Lp (Watts and Strogatz, 1998). The Graph theoretical network analysis (GRETNA) toolbox (Wang et al., 2015) was utilized to study the topological characteristics of the brain network at the global and node levels. In addition, the global efficiency (Eglob) quantified the information transmission capacity of the whole brain network, and the local efficiency (Eloc) quantified the information transmission capacity of a single node (Latora and Marchiori, 2001).

### Network Modularity

All non-zero elements from the structure matrix of each participant were extracted, and then the sparsity-based threshold method was applied to calculate SC-FC coupling. Specifically, sparsity was defined as the percentage of actual connections to the number of possible connections in the network. We evaluated coupling by applying a 30% sparsity threshold to define functional connectivity. In addition, FS coupling was calculated using the Spearman rank correlation between functional connectivity and their non-zero structural connectivity counterparts at the whole brain level (correlation of the whole brain) and each Module level (correlation within the Module; Collin et al., 2017; Fukushima et al., 2018; Baum et al., 2020). Consistent with research studies, Module I (red) is responsible for cognitive control. Module II (yellow) is related to the execution of cognitive processing. Module III (green) corresponds to sensorimotor, auditory, and language functions. Known Module IV (blue) involves vision and language functions. Finally, Module V (Navy Blue) is related to emotional processing (Buckner et al., 2009).

### Connection Strength Within Modules

The connection strength within the Module was an evaluation index of the importance of a specific Module in the brain network, which was calculated as the average of all connection weights in the Module. The inter-Module connection strength between two Modules represented the average of the connection weights connecting the two Modules. The current research calculated the connection strength within and between Modules in the SC and FC network.

### Statistical Analysis

All non-zero elements from the structure matrix of each participant were extracted, and then we use the sparsity-based threshold method to calculate the SC-FC coupling. Specifically, sparsity was defined as the percentage of actual connections to the number of possible connections in the network. We evaluated coupling by applying a 30% sparsity threshold to define FC (Ma et al., 2021). In addition, Spearman rank correlation was calculated using SC and FC at the whole brain level (correlation of the whole brain) and each Module level (correlation within the Module; Collin et al., 2017; Fukushima et al., 2018; Baum et al., 2020). The connection strength in the Module was defined as the average value of all connection weights in the Module.

SPSS 23.0 software was utilized for the statistical analysis of demography and behavior. Chi-square test was used for categorical variables and independent samples *t*-tests for continuous variables. The non-parametric permutation test (1,000 permutations) was used to evaluate the difference between the whole brain and modular SC-FC coupling and the connection strength with the Module. Multiple comparisons used the false discovery rate (FDR), and *P* < 0.05 was statistically significant. In addition, we used gender, age, and years of education as confounding variables to analyze the correlation between each Module and depression factors using multiple linear regression methods.

## Results

### Demographic Characteristics and Clinical Symptoms

There were no significant differences in the basic data (i.e., age, sex, education, duration) and functional assessment scores (i.e., MMSE, MoCA, and NIHSS scores) between the PSD and non-PSD groups (*p* > 0.05). Detailed demographics were listed in Table 1.

**Table 1:** Demographic details of the recruit participants in this study.

		PSD	Non-PSD	*P*-value
Number of subjects	(Male/Female)	23 (11/12)	22 (10/12)	0.6547^a^
Education (years)	Mean ± std. dev.	7.5 ± 4.4	6.8 ± 4.3	0.063^b^
BMI (years)	Mean ± std. dev.	75.6 ± 13.8	73.1 ± 21.0	0.29^b^
MMSE	Mean ± std. dev.	27.6 ± 2.2	24.7 ± 3.8	0.074^b^
MoCA	Mean ± std. dev.	26.9 ± 2.9	27.6 ± 5.1	0.054^b^
Age (years)	Mean ± std. dev.	73.1 ± 5.7	71.2 ± 8.3	0.324^b^
Duration(months)	Mean ± std. dev.	2.8 ± 2.5	3.2 ± 2.8	0.61^b^
NIHSS score	Mean ± std. dev.	1.2 ± 1.4	2.1 ± 2.2	0.06^b^
HAMD score	Mean ± std. dev.	26.2 ± 3.8	13.3 ± 5.1	<0.001^b^
MoCA	Mean ± std. dev.	27.5 ± 1.5	19.7 ± 5.1	<0.01
Anxiety/Somatization	Mean ± std. dev.	4 ± 3.2	3 ± 2.6	0.053
Weight loss	Mean ± std. dev.	1 ± 0.4	1 ± 0.4	0 0.276^b^
Cognitive dysfunction	Mean ± std. dev.	8 ± 3.2	1 ± 2.6	<0.001^b
Atypical circadian rhythm	Mean ± std. dev.	1 ± 3.2	1 ± 2.6	0.68
Sleep Disorder	Mean ± std. dev.	2 ± 2.2	2 ± 2.6	0.56
Retardation	Mean ± std. dev.	6 ± 3.2	1 ± 2.1	<0.001^b^
Desperation	Mean ± std. dev.	3 ± 2.7	2 ± 3.4	0.019

*BI, Barthel Index; MMSE, Mini Mental State Examination; NIHSS, National Institutes of Health Stroke Score; std. dev, standard deviation. ^a^The p values are obtained by using *χ*^2^ test. ^b^The p values are obtained by using a two-sample *t*-test. *p* < 0.05 is considered significant*.

### The SW Attributes and Network Topology

**Figure 2:**
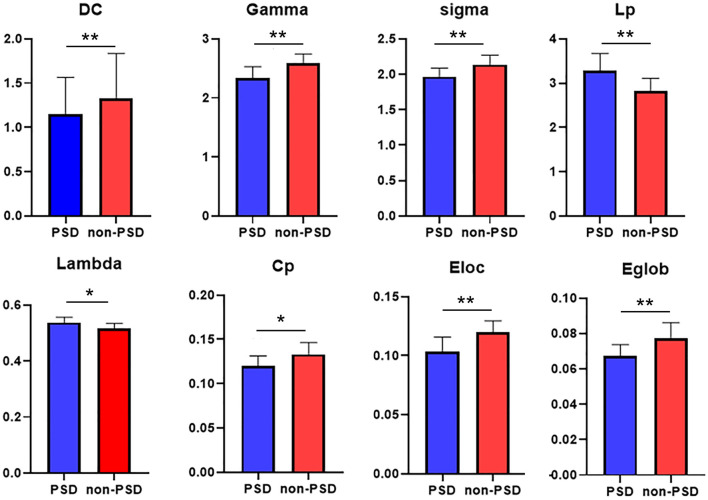
Between group comparisons of global network measures. DC (degree coefficient), clustering coefficient (Cp), feature path length (Lp), normalized clustering coefficient (γ), standard feature path length (λ), small-world attribute (σ), global efficiency (Eglob), and local efficiency (Eloc; *p* < 0.05). The difference was statistically significant. *p < 0.05, **p < 0.01. The color bar represents the Module and the size represents the weight of node.

Both the PSD group and the non-PSD group had SW attributes, while the σ of the PSD group was smaller than the non-PSD group (*p* < 0.05). In addition, the degree coefficient (DC), γ, Cp, Eloc, and Eglob of the PSD group were lower than those of the non-PSD group, while the λ and Lp showed the opposite trend (*p* < 0.05; Figure 2).

**Figure 3:**
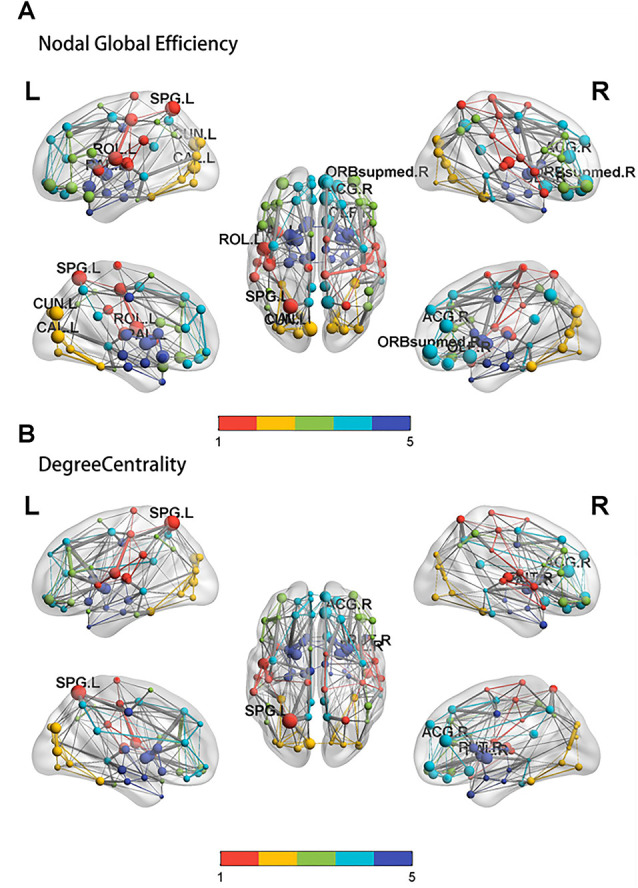
The results of the nodal global efficiency and DC. Compared with controls, the result of the nodal global efficiency **(A)** and DC **(B)** in the PSD group. SPG: superior parietal lobule, ACG: anterior cingulated gyrus, ORB supmed: Superior frontal gyrus, medial orbital, CAL: calcarine, CUN: cuneus, OLF: olfactory cortex, ROL: Rolandic operculum, PAL: pallidum.

The nodal global efficiencies of the orbit frontal cortex, superior parietal lobule, cuneus, precentral gyrus, olfactory cortex, pallidum, calcarine, and anterior cingulate gyrus in the PSD group were lower than the non-PSD group (*p* < 0.05; Figure 3A). Compared with non-PSD, DC was reduced in the brain area including superior parietal lobule, anterior cingulated gyrus, putamen, pallidum in the PSD group (*p* < 0.05; Figure 3B; the color bar represents the Module and the size represents the weight of the node).

### Modular Organizations

**Figure 4:**
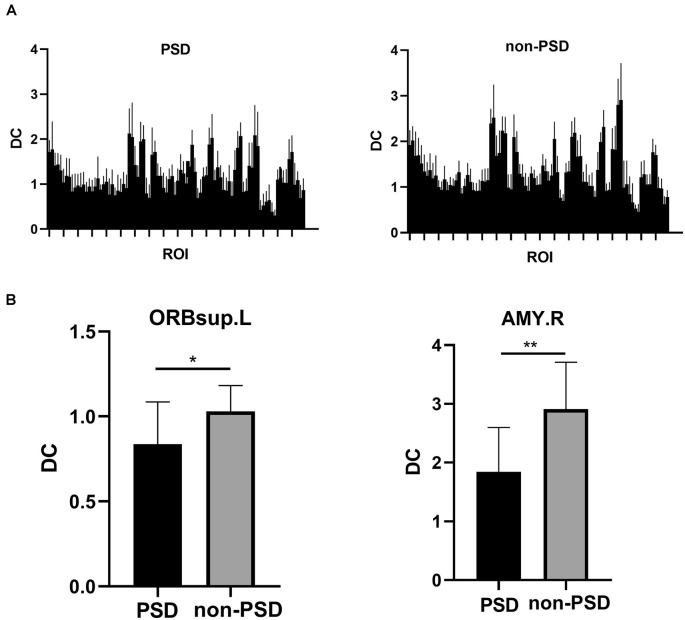
**(A)** The DC differences between 90 regions of interest (ROIs) of the AAL template in the the PSD and the non-PSD groups. **(B)** The region with decreased DC in the PSD group was located in the left superior orbital frontal gyrus and the right amygdala (*p < 0.05, **p < 0.01, FDR correction).DC: Degree centrality. ORBsup: superior orbital frontal gyrus; AMY: amygdala; L: left; R: right.

There was a significant difference in whole brain modularity between PSD patients and non-PSD patients (*p* = 0.039, *t* = 0.35, FDR corrected; Figure 4A). As shown in Figure 4B, compared with non-PSD group, decreased DC in PSD was located in the left superior orbital frontal gyrus (*p* = 0.0286, *t* = 2.343, FDR corrected) and the right amygdala (*p* = 0.0029, *t* = 3.353, FDR corrected).

**Figure 5:**
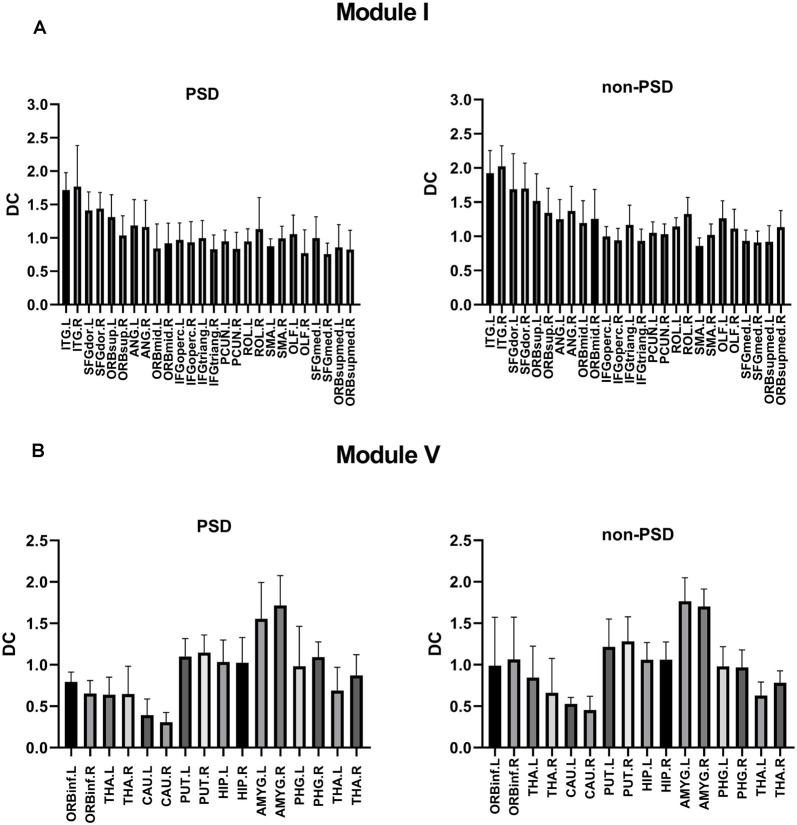
**(A)** Comparison of the DC values of Module I in the PSD group and the non-PSD group (*p* = 0.029, *t* = 1.456, FDR correction). **(B)** Comparison of the DC values of Module V in the PSD group and the non-PSD group (*p* = 0.038, *t* = 2.126, FDR correction).

The DC values of Module I and Module V were different between PSD and non-PSD groups (*p* = 0.029, *t* = 1.456, FDR corrected; *p* = 0.038, *t* = 2.126, FDR corrected, respectively; Figure 5).

### Associations Between Global-Wise Metrics and Depression Performance

**Figure 6:**
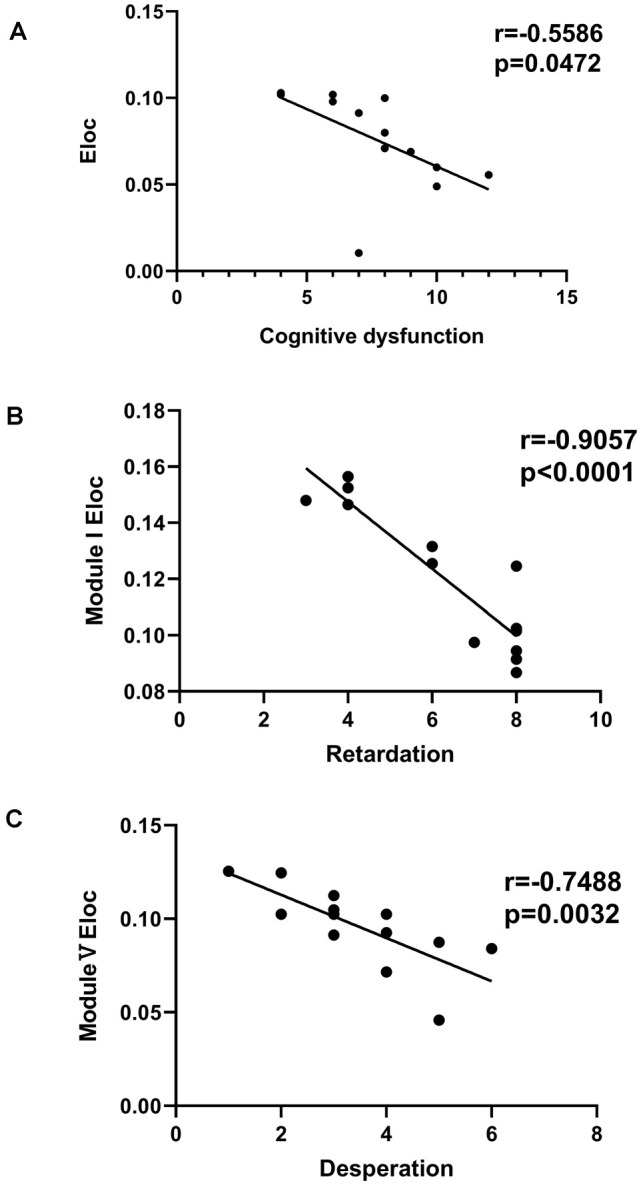
Correlation analysis between clinical variables and global efficiency in the PSD group. **(A)** There was a negative correlation between the nodal global efficiency and the cognitive dysfunction score. **(B)** In Module I, there was a negative correlation between the nodal global efficiency and the retardation score. **(C)** The nodal global efficiency in Modules V showed a negative correlation with the desperation score.

Significant differences in cognitive dysfunction, retardation and desperation were found by factor analysis (*p* < 0.05, FDR corrected). There was a negative correlation between Eloc and the cognitive dysfunction scores (*r* = −0.5586, *p* = 0.0472, FDR corrected; Figure 6A). In Module I, there was a negative correlation between Eloc and the retardation scores (*r* = −9057, *p* < 0.001, FDR corrected; Figure 6B). Besides, Eloc in Modules V showed a negative correlation with the desperation score (*r* = −0.7488, *p* = 0.0032, FDR corrected; Figure 6C).

### Group Differences in SC-FC Coupling

**Figure 7:**
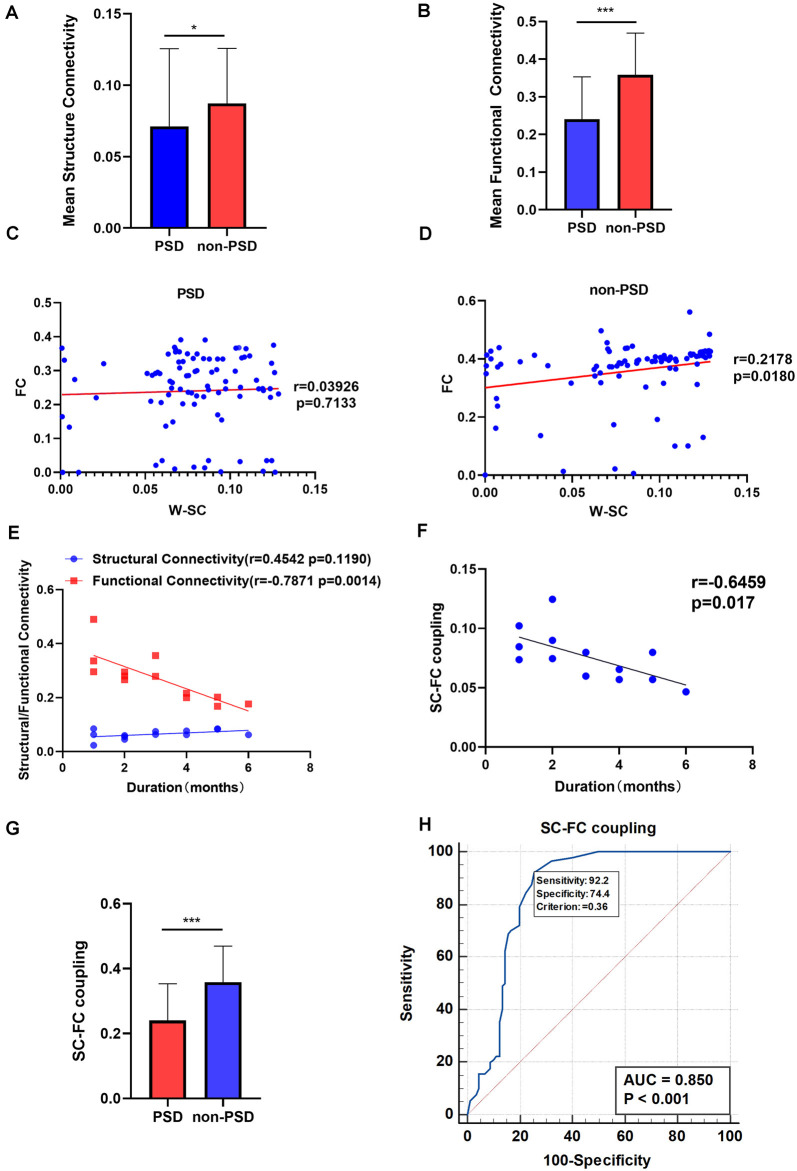
**(A)** The mean SC in the PSD and the non-PSD groups. **(B)** The mean FC in the PSD and the non-PSD groups. **(C)** The SC-FC coupling in the PSD group. **(D)** The SC-FC coupling in non-PSD group. **(E)** Correlation between the mean SC/ FC and duration of PSD. **(F)** Correlation between the SC-FC coupling and duration of PSD. **(G)** The SC-FC coupling in the PSD and the non-PSD groups. **(H)** A receiver operating characteristic (ROC) curve for PSD and non-PSD. *p < 0.05, ***p < 0.001. SC, structural connectivity; FC, functional connectivity; W-SC, weighted SC.

Both the mean SC and mean FC of the whole brain in patients with PSD were lower than that in non-PSD patients after stroke (*p* = 0.0238, FDR corrected; *p* < 0.0001, FDR corrected, respectively; Figures 7A,B). Correlation coefficients between SC and FC in PSD group and non-PSD group were *r* = 0.03926 (*p* = 0.7133, FDR corrected), *r* = 0.2178 (*p* = 0.0180, FDR corrected), respectively, which meant PSD had a lower SC-FC coupling than non-PSD (Figures 7C,D). The mean FC was negatively correlated with the duration of PSD (within 0–6 months; *r* = −0.7871, *p* = 0.0014). However, there was no correlation between the mean SC and the duration of PSD (Figure 7E). In addition, we also found a negative correlation between the PSD duration and the SC-FC coupling (within 0–6 months; *r* = −0.6459, *p* = 0.017; Figure 7F). Generally, the SC-FC coupling in the PSD group was lower than that in the non-PSD group (*p* < 0.001; Figure 7G). Moreover, a receiver operating characteristic (ROC) curve was plotted to investigate whether SC-FC coupling could distinguish PSD from non-PSD patients. As shown in Figure 7H, SC-FC coupling correctly classified 17 of 23 PSD patients and 20 of 22 non-PSD, with a sensitivity and specificity of 92.22% and 74.44% respectively. The area under the curve for the ROC was 0.85 (95% confidence intervals from 0.79 to 0.90), indicating that SC-FC coupling could be used as a potential marker for the diagnosis of PSD.

## Discussion

In this study, we used graphical theoretical analysis to investigate the network topology and the coupling between SC and FC networks in stroke patients. This is the first study about the brain Module and its relationship with depressive symptoms. The abnormal nodes are important causes of cognitive control and emotional processing. Our results showed that the decrease in node efficiency (the superior orbital frontal gyrus, anterior cingulated gyrus, and amygdala) lead to the patients’ core symptoms such as dullness and despair. In addition, many regions were altered in the SC network, involving attention, sensory motion, subcortical and default mode networks. Most importantly, we found that the Module level was abnormal and the SC-FC coupling was significantly reduced in PSD patients, which may be helpful to diagnose depression in stroke patients. The potential use of multimodal neuroimaging biomarkers for PSD is highlighted.

### Modular Organization

Module I is responsible for cognition control and Default Mode Network (DMN) functions. Due to the decreased coefficient of Module I, PSD patients may not be able to effectively adjust their thoughts and behaviors. The results of this study showed that the mean value of the nodal efficiency of Module I was negatively correlated with the retardation score of HAMD. Cognition impairment may also be related to the network of Module I in PSD patients. In PSD patients, Module I appeared to be responsible for DMN, while cognitive control and strategy/executive functions are managed jointly by Module I and Module II. By extension, the cognition of helplessness may then be related to the structural network of Module I among PSD patients.

### Altered Regional Topology of SC and FC Networks

Both the nodal efficiency and DC may contribute to the alteration of Module organization. Our study found that decreased DC in PSD was mainly located in the left superior orbital frontal cortex and the right amygdala. Emotional processing receives inputs from the amygdala that encodes emotional valence, and motivation-related inputs from the orbitofrontal cortex that is involved in binding stimulus to response (Haber et al., 2006). In our study, the decreased DC was found in the DMN region of the PSD patient, and we further confirmed the separation of the DMN in Module I. Similarly, previous studies reported that decreased FC between the amygdala and prefrontal cortex was the basis for fear, anxiety, and depression (Prater et al., 2013). The abnormalities were found in Module I and Module V, which may be responsible for the impairments of cognitive processing (Module I function) and emotional processing (Module V function) and be related to the clinical symptoms such as dullness and helplessness.

Compared with the non-PSD group, patients in the PSD group showed altered topological patterns: reduced clustering coefficients and increased path lengths. Our results suggest that the balance between SC and FC networks is disrupted in PSD patients. At present, the rate of misdiagnosis of PSD is very high, delaying treatment has serious consequences for patients, and it is important to distinguish whether patients are depressed after a stroke. Previous studies have shown that the brain network in PSD patients tends to be more randomized than non-PSD. In this study, we found a decrease in DC and node efficiency in PSD patients, and this finding may provide new neuroimaging markers to help diagnose PSD.

The orbital frontal cortex is related to working memory and mood (Ray and Zald, 2012). Therefore, reduced node efficiency in the area may support the hypothesis that depression is associated with orbitofrontal lesion altering brain dynamics of emotion-attention and emotion-cognitive control interaction in humans (Kuusinen et al., 2018).

The reduction of node efficiency was also found in some areas (ROL) belonging to the sensory-motor network. Sensory-motor network is related to self-starting movement and unconscious movement inhibition.

In addition, some brain regions (PHG, SFG), parts of the default mode network, also exhibited reduced node efficiency. Previous studies showed that DMN damage was associated with depression (Raichle et al., 2001). Our findings also support the damage to the default network of PSD patients.

The increased path length region is primarily related to the subcortical regions (AMYG, CAU, PUT, and HIP). The subcortical region plays an important role in the regulation of mood and cognition (Haber et al., 2006). Impairment in the subcortical region will result in executive dysfunction, memory recovery deficits, and uncontrolled emotions, which is consistent with clinical manifestations in patients with PSD.

In this study, we found that the connectivity of functional networks changed more than that of structural networks, which is not surprising since there are studies reporting that network nodes are more susceptible to FC disorders due to changes in neurotransmitter levels (Skudlarski et al., 2008).

### Decoupling Between SC and FC Networks

This study investigated the SC-FC coupling in large-scale brain networks between PSD and non-PSD patients. The functional network represents the temporal consistency of brain regions, while the structural network reflects the anatomical integrity of white matter regions and is the basis of communication between brain regions (Noonan et al., 2018). SC is highly predictive and restrictive for FC. In contrast, FC exerts an effect on SC through plasticity mechanisms (Hagmann et al., 2010), which indicates a complex relationship between SC and FC. The split-half reliability analysis confirmed the stability and reliability of the results of this study.

As we predicted, SC-FC coupling in PSD patients was reduced, reflecting the abnormal mechanism of the brain network of PSD patients. Specifically, *via* using coupling intensity as an indicator, we can distinguish PSD patients from non-PSD, which is potentially useful for improving the diagnosis and evaluation of PSD.

Our study showed some regional characteristics were altered in the SC network, involving attention, sensorimotor, subcortical, and default-mode networks.

### Strengths and Limitations

First, the patient sample size is relatively small. However, even in this small group, statistically significant changes in connectivity were found. Secondly, an SC and FC network was established at the network level, including 90 brain regions from the AAL brain map. There are also different spatial scales that may show different network topology organizations. Thirdly, it is difficult to explain the causal relationship between the SC and FC network, which needs to be further studied in combination with dynamic connection analysis. Finally, the potential influence of different scales on the assessment of depressive symptoms deserves further investigation.

## Conclusions

We have distinguished PSD patients from non-PSD patients by SC-FC coupling, which suggests that the coupling intensity of SC and FC may be an important feature involved in the PSD mechanism. Moreover, our findings imply that the SC may be the key determinant of brain dysfunction, and SC-FC coupling could serve as a potential biomarker for PSD. Such a measure may have clinical significance for the early identification of PSD and inform strategies for prevention. This study emphasizes the importance of multimodal brain connectors for a better understanding of PSD.

## Data Availability Statement

The original contributions presented in the study are included in the article, further inquiries can be directed to the corresponding author.

## Ethics Statement

The studies involving human participants were reviewed and approved by Ethics Committee of the Zhujiang Hospital of Southern Medical University, China. The patients/participants provided their written informed consent to participate in this study.

## Author Contributions

Study concept and design: XFZ and WW. Acquisition, analysis, interpretation of data: XFZ, YS, and TF. Drafting of the manuscript: XFZ and WW. Critical revision of the manuscript: YS, KLW, HRZ, and WW. Statistical analysis: XFZ. MRI technical support: KLW. Study supervision: WW. All authors contributed to the article and approved the submitted version.

## Conflict of Interest

The authors declare that the research was conducted in the absence of any commercial or financial relationships that could be construed as a potential conflict of interest.

## Publisher’s Note

All claims expressed in this article are solely those of the authors and do not necessarily represent those of their affiliated organizations, or those of the publisher, the editors and the reviewers. Any product that may be evaluated in this article, or claim that may be made by its manufacturer, is not guaranteed or endorsed by the publisher.

## Footnotes

^1^ https://www.nitrc.org/projects/panda/

## Abbreviations

PSD: post-stroke depression
non-PSD: post-stroke without depression
FA: fractional anisotropy
MD: mean diffusivity
SC: structural connectivity
FC: functional connectivity
DTI: diffusion tensor imaging
rs-fMRI: resting-state functional magnetic resonance imaging
SW: small-world
NIHSS: National Institutes of Health Stroke Score
MMSE: Mini-Mental State Examination
BI: Barthel Index
HAMD: Hamilton Depression Scale
DSM-V: Diagnostic and Statistical Manual of Mental Disorders, Fifth Edition
TR: repetition time
TE: echo time
MNI: Montreal Neurological Institute
FWHM: full width at half maximum
WM: white matter
DPARSF: Data Processing Assistant for Resting-State fMRI
BOLD: blood oxygen level-dependent
FDR: false discovery rate
TFCE: Threshold-Free Cluster Enhancement
ss-EPI: single-shot echo-planar imaging
AAL: automated anatomical labeling
GLM: general linear modeling
DC: degree coefficient
Cp: clustering coefficient
Lp: feature path length; γ, normalized clustering coefficient; λ, standard feature path length, σ, small-world attribute value
Eglob: global efficiency
Eloc: local efficiency
AUC: the area under the curve
ROI: region of interest
DMN: Default Mode Network
PreCG: Precental gyrus
HP: hippocampus gyrus
PHP: parahippocampa gyrus
ACG: anterior cingulate cortex
PUT: putamen
CAL: calcarine
FFG: fusiform gyrus
ROL: Rolandic_OperL
SFG: superior frontal gyrus
AMYG: amygdala.
